# Deposition of C-terminally truncated Aβ species Aβ37 and Aβ39 in Alzheimer’s disease and transgenic mouse models

**DOI:** 10.1186/s40478-016-0294-7

**Published:** 2016-03-08

**Authors:** Jochim Reinert, Bernhard C. Richard, Hans W. Klafki, Beate Friedrich, Thomas A. Bayer, Jens Wiltfang, Gabor G. Kovacs, Martin Ingelsson, Lars Lannfelt, Anders Paetau, Jonas Bergquist, Oliver Wirths

**Affiliations:** Division of Molecular Psychiatry, University Medical Center (UMG), Georg-August-University, Göttingen, Germany; Department of Psychiatry and Psychotherapy, University Medical Center (UMG), Georg-August-University, von-Siebold-Str. 5, 37075 Göttingen, Germany; Synaptic Systems, Göttingen, Germany; Institute of Neurology, Medical University of Vienna, Vienna, Austria; Department of Public Health and Caring Sciences, Rudbeck Laboratory, University of Uppsala, Uppsala, Sweden; Department of Pathology, University and University Hospital of Helsinki, Helsinki, Finland; Analytical Chemistry, Department of Chemistry – Biomedical Centre and SciLifeLab, Uppsala University, Uppsala, Sweden

**Keywords:** Alzheimer, C-terminal truncation, Amyloid precursor protein, Transgenic mice, Aβ37, Aβ39, Immunohistochemistry, Mass spectrometry

## Abstract

In Alzheimer’s disease (AD) a variety of amyloid β-peptides (Aβ) are deposited in the form of extracellular diffuse and neuritic plaques (NP), as well as within the vasculature. The generation of Aβ from its precursor, the amyloid precursor protein (APP), is a highly complex procedure that involves subsequent proteolysis of APP by β- and γ-secretases. Brain accumulation of Aβ due to impaired Aβ degradation and/or altered ratios between the different Aβ species produced is believed to play a pivotal role in AD pathogenesis. While the presence of Aβ40 and Aβ42 in vascular and parenchymal amyloid have been subject of extensive studies, the deposition of carboxyterminal truncated Aβ peptides in AD has not received comparable attention. In the current study, we for the first time demonstrate the immunohistochemical localization of Aβ37 and Aβ39 in human sporadic AD (SAD). Our study further included the analysis of familial AD (FAD) cases carrying the APP mutations KM670/671NL, E693G and I716F, as well as a case of the PSEN1 ΔExon9 mutation. Aβ37 and Aβ39 were found to be widely distributed within the vasculature in the brains of the majority of studied SAD and FAD cases, the latter also presenting considerable amounts of Aβ37 containing NPs. In addition, both peptides were found to be present in extracellular plaques but only scarce within the vasculature in brains of a variety of transgenic AD mouse models. Taken together, our study indicates the importance of C-terminally truncated Aβ in sporadic and familial AD and raises questions about how these species are generated and regulated.

**Electronic supplementary material:**

The online version of this article (doi:10.1186/s40478-016-0294-7) contains supplementary material, which is available to authorized users.

## Introduction

Amyloid-β (Aβ) peptides have been at the center of Alzheimer’s disease (AD) research since their identification as the main component of extracellular plaques within the brains of AD patients [13, 28]. The amyloid hypothesis states that an imbalance in the production and clearance of Aβ initiates a cascade of pathological events, including the hyperphosphorylation of tau, that ultimately results in neuron loss [17]. Aβ peptides were found to bear different characteristics depending on their C-terminus, with Aβ42 proven to be extraordinarily prone to aggregation [22]. At present, there is growing evidence that even subtle changes in the spectrum of Aβ peptides, i.e. an increase of the ratio of Aβ42/40, may facilitate AD pathogenesis, presumably through the formation of toxic Aβ oligomers [16, 25].

Providing strong support for the amyloid cascade hypothesis, rare point mutations within the amyloid precursor protein (APP) and presenilin (PSEN) genes have been found to cause autosomal-dominant inheritance in FAD [50]. Mechanisms by which FAD related mutations can cause the disease include (i) an increased overall Aβ production, (ii) a change in aggregation behavior of Aβ and (iii) a shift in the Aβ peptide spectrum produced [4, 15]. Conclusions drawn from the study of these mechanisms may eventually translate into successful treatment of the by far more common sporadic form of AD (SAD).

Aβ peptides are generated by sequential processing of the single-transmembrane APP. In this process, the γ-secretase, consisting of a complex containing PSEN among other components, has been found to determine the C-terminus of the peptide [3]. Its action is preceded by β-secretase cleavage, releasing the soluble N-terminal ectodomain (sAPPβ) and leaving a short membrane-bound APP C-terminal fragment (β-CTF, C99) [35]. Current models describe the underlying mechanism by which γ-secretase generates Aβ of varying length as a stepwise cleavage of β-CTF APP, in which the initial ε-cleavage critically affects the outcome [9] with cleavage at either T48 or L49, resulting in subsequent production of Aβ42 or Aβ40, respectively [6]. It has been proposed that the products of ε-cleavage – Aβ49 and Aβ48 – are further processed by subsequent γ-secretase cleavages at every 3 to 4 residues, so that two major product lines emerge: Aβ49 > Aβ46 > Aβ43 > Aβ40 and Aβ48 > Aβ45 > Aβ42 > Aβ38. This hypothesis is based on the in vitro detection of corresponding tri- and tetrapetides [43]. Remarkably, several deviations from this scheme, like the independent generation of Aβ38 from Aβ42, have been reported [8, 39]. A recent study by Matsumura et al. suggests a more complicated picture in which γ-secretase action occasionally cleaves at every fourth, fifth or even sixth residue, thereby interlinking the two major product lines [29]. In addition, the production of further Aβ species including Aβ37 and Aβ39 was demonstrated: The release of the tripeptides GVV and VIA corresponds to the generation of Aβ37 from Aβ40 and of Aβ39 from Aβ42 respectively. Furthermore, Aβ37 can also originate directly from Aβ42, by release of the GVVIA pentapeptide [29].

So called γ-secretase modulators (GSMs) present a pharmaceutical approach to change the Aβ spectrum produced by γ-secretase, while not affecting other physiological functions of the enzyme complex [12]. GSMs of the non-steroidal anti-inflammatory drug-type have been shown to specifically decrease levels of Aβ42 in vitro, partially by shifting the production to C-terminally truncated Aβ [3, 54]. Very recently, peptide inhibitors based on the hexapeptide fragment Aβ_32-37_ have been generated. These inhibitors showed significant Aβ aggregation inhibitory activity and mitigation of Aβ toxicity, underscoring the importance of Aβ C-terminal truncations [2].

While a plethora of studies have focused on the deposition of the most abundantly produced species Aβ40 and the presumably most toxic species Aβ42, the C-terminally truncated species Aβ37, Aβ38 and Aβ39 have received less attention. All three species have been reported to be present in cerebrospinal fluid (CSF) [40, 55] and human plasma [27] and might be of importance to increase diagnostic accuracy when using CSF samples [47], but immunohistochemical analysis for C-terminally truncated Aβ species have so far only focused on Aβ38. For this species, Moro et al. and our group recently reported abundant deposition in the vasculature in sporadic AD (SAD) cases presenting severe cerebral amyloid angiopathy (CAA), as well as within NP and vascular amyloid deposits of different FAD cases [32, 44]. Our present study extends these findings by the analyses of the deposition of Aβ37 and Aβ39 in SAD, FAD and common animal models of the disease. Very much like Aβ38, we found both Aβ37 and Aβ39 to be detectable within the vasculature of the majority of SAD cases investigated. In addition, we show the presence among four FAD cases carrying either APP or PSEN1 mutations. This includes the analysis of a recently described novel APP mutation I716F [14]. Moreover, we found the C-terminally truncated Aβ species to be deposited as plaques within a variety of established transgenic mouse models of AD, including APP23 and 5XFAD among others.

## Material and methods

### Patients

We examined the brains of sporadic AD cases (*n* = 13; age: 88.5 ± 4.2 years), AD with CAA (*n* = 2), Down syndrome (DS, *n* = 3) and several different familial AD cases (*n* = 4) in comparison to non-demented control patients (NDC) (*n* = 8; age: 80.5 ± 7.2 years). The mutations underlying FAD were the APP mutations KM670/671NL (Swedish), E693G (Arctic) [23], I716F [46] and the PSEN1 mutation ΔExon 9 [53]. Human brain samples were obtained from the following sources: Netherlands Brain Bank, University Hospital Helsinki, Medical University Vienna and Uppsala University. Definite diagnoses were based on established criteria and written informed consent had been received from all subjects or their close relatives, according to ethical regulations in each country.

### Animal models of AD

Formalin-fixed and paraffin embedded brain tissue was obtained from six transgenic mouse lines that are commonly used as AD models, namely PDAPP [10], APP23 [48], 3xTg [37], APP/PS1ΔEx9 [11], 5xFAD [36] and APP/PS1KI [5] (Table 3).

### Immunohistochemistry

Immunohistochemistry was performed on 4 μm sagittal paraffin sections, as previously described [57]. In brief, sections were deparaffinized in xylene and rehydrated using an ascending series of ethanol (70 %, 95 %, and 100 %). Endogenous peroxidases were blocked by incubation in 0.3 % H_2_O_2_ in 0.01 M PBS. Antigen retrieval was achieved by boiling in 0.01 M citrate puffer and 3 min incubation in 88 % formic acid (FA). Prior to incubation with primary antibodies, blockage of non-specific binding sites was secured by treatment with 4 % skim milk and 10 % fetal calf serum in 0.01 M PBS for 1 h at ambient temperature. Mouse monoclonal antibodies 4G8 (Covance, Dedham) against Aβ, G2-10 (Milipore, Schwalbach) against Aβ40, 326 F1 against Aβ38 (#218421, Synaptic Systems, Göttingen), rabbit monoclonal antibodies mAb12467 against Aβ37 and mAb12077 against Aβ39 (both Cell Signaling) and a rabbit polyclonal antibody against Aβ42 (#218703, Synaptic Systems, Göttingen) were incubated overnight at ambient temperature. Incubation with biotinylated secondary antibodies (DAKO, Glostrup, 1:200) was carried out at 37 °C and was followed by applying the ABC method with a Vectastain kit (Vector Laboratories, Burlingame, USA) and diaminobenzidine as a chromogen to reveal the staining. Hematoxylin was used for counterstaining. Double-immunofluorescence staining was performed using DyLight488 and DyLight594 fluorescent secondary antibodies (Thermofisher Scientific). Counterstaining was performed with 4’6-diamidine-2’phenylindole dihydrochloride (DAPI, Sigma-Aldrich, Taufkirchen).

### Mass spectrometry

#### Formic acid extraction of proteins from mouse brain

Three left brain hemispheres from 7-month-old female 5XFAD mice were homogenized to powder in liquid nitrogen. Aliquots of ~50 μg brain powder were re-suspended in 300 μl PBS pH 7.4 supplemented with cømplete protease-inhibitor (Roche, 1 tablet/10 ml) and further sonicated using an ultrasound-130 Watt ultrasonic processor Sonics Vibra-Cell VCX-130 (Sonics & Materials, Newtown, USA) (Ampl. 30 %, Pulse 2, 1 min). Extraction was carried out by adding 660 μl FA and sonicating again for 1 min. The extract was spun down for 20 min at 17.000 x g and 4 °C. Following determination of total protein concentration using the DC Protein Assay Kit, aliquoted extracts were dried down using a SpeedVac at 45 °C.

#### Immunoprecipitation of Aβ and preparation of extracts for MALDI-TOF

Immunoprecipitation was carried out with monoclonal anti-Aβ antibodies (1:1 mixture of antibodies 6E10/4G8 (Covance) coupled to paramagnetic Dynabeads M-280 sheep-anti-mouse. In brief, 20-fold diluted brain extract neutralized with 1 M Tris base, 0.5 M Na_2_HPO_4_ was incubated with functionalized Dynabeads (8 μg antibody/50 ml beads) on a rotator for 6 h at 4 °C. After incubation with the sample, beads were washed twice with PBS pH 7.4 supplemented with 0.1 % BSA, and twice in 50 mM ammonium bicarbonate. After washing, elution of precipitated peptides was performed by incubation of the beads with 100 μl 0.5 % FA under vortexing for 15 min. The eluates were finally aliquoted and dried at 45 °C in a SpeedVac and stored at -80 °C. Immediately before MALDI-TOF-MS analysis, the dried eluate obtained from IP was dissolved in 20 μl of 20 % acetonitrile, 0.1 % FA and sonicated in an ultrasonic water bath for 10 min.

#### MALDI-TOF-MS analysis

Samples and standards were plated at 1 μl with an equal amount of sinapinic acid (SA) (20 mg/ml in 1:1 (v/v) mixture of acetonitrile/water) and left to dry at room temperature. For calibration, we used 1 μl synthetic Aβ peptides (pyroglutamate Aβ_pE3-40/42_ and Aβ_4-40/42_; dissolved at 0.01 mg/ml each in 10 mM NaOH). The MALDI-TOF-MS experiments were performed on a Bruker Daltonics MALDI Ultraflex II spectrometer equipped with a pulsed N_2_ laser (337 nm) in a positive reflector mode with delayed extraction (150 ns). The level of laser power was adjusted before each experimental session to allow for sufficient ionization and to avoid saturation of MS detector. Spectra were acquired automatically for the m/z range of 2000 – 6000 with each spectrum being the sum of 10,000 single laser shots. The following Instrument settings were chosen : Ion source I 25.0 kV, ion source II 21.7 kV, lens voltage 10.1 kV, reflector voltage I 26.3 kV, reflector voltage II 13.8 kV, laser repetition rate 66 Hz.

### Urea SDS-PAGE and Western-immunoblot

One-dimensional urea-Bicine/Bis-Tris/Tris/sulfate SDS-PAGE (1D-Aβ-PAGE) was carried out as described previously [45]. Protein extracts were prepared from 12-month-old WT and 5XFAD mouse brain hemispheres by sequential extraction with Tris-buffered saline (TBS, 120 mM NaCl, 50 mM Tris, pH7.5) and 2 % sodium dodecyl sulfate (SDS). In brief, brain hemispheres were homogenized in TBS in a weight:volume ratio of 1:10 using a glass Teflon homogenizer. Following centrifugation for 20 min at 17000 x g, the resulting pellet was sonified in 2 % SDS and centrifuged for 20 min at 17000 x g. The protein concentrations of the supernatants were determined with the Roti-Quant protein assay (Carl Roth). Aβ peptide immunoprecipitation was carried out with mAb6E10 (Covance) as previously described [18, 45]. For Western-immunoblot analysis, samples with a protein concentration of 2 mg/mL were prepared in electrophoresis sample buffer (final composition: 0.36 M Bistris, 0.16 M bicine, 15 % (w/v) sucrose, 1 % (w/v) SDS, 0,0075 % bromophenol blue). Of each sample, 10 μL (20 μg of total protein) were separated on a 10 % T / 5 % C urea Bicine/Bis-Tris/Tris-sulfate SDS-polyacrylamide gel and blotted onto a PVDF membrane for 45 min at 1 mA /cm2 with a discontinuous buffer system, essentially as described in [56]. The blot sandwich was assembled from the anode (+) to the cathode (-) by stacking 1 filter paper (extra thick blot paper, Biorad) soaked in 0.21 M Tris / 30 % methanol, 1 filter paper soaked in 25 mM Tris / 30 % methanol, the PVDF-membrane preequilibrated in 25 mM Tris / 30 % methanol, the polyacrylamide gel briefly pre-incubated in 25 mM Tris-borate, pH 9.0 / 0.025 % SDS and 2 filter papers soaked with 25 mM Tris-borate, pH 9.0 / 0.025 % SDS. After the electrophoretic transfer, the PVDF membranes were boiled for 3 min in PBS in a microwave oven to facilitate the immunodetection [21]. The blots were blocked overnight at 4 °C with 2 % GE-block in PBS-T (PBS with 0.075 % (v/v) Tween-20) and subsequently probed with mAb1E8 for 1 h at room temperature: After 3 x 10 min washing with PBS-T, the biotinylated secondary anti-mouse IgG antibody (Linaris) was applied for 45 min. After 3x washing with PBS-T, the blot was incubated with streptavidin-coupled horseradish peroxidase for 45 min at room temperature [18]. Following 3 x 10 min washing with PBS-T, the blots were developed with ECL-prime (GE-Healthcare) for 5 min at room temperature, and the signals were recorded with a LiCor imager. A mixture of the synthetic Aβ peptides (Aβ1-37, Aβ1-38, Aβ1-39, Aβ1-40, Aβ1-42) served as a reference for the electrophoretic mobility of the different Aβ variants.

## Results

### Sporadic AD

**Fig. 1:**
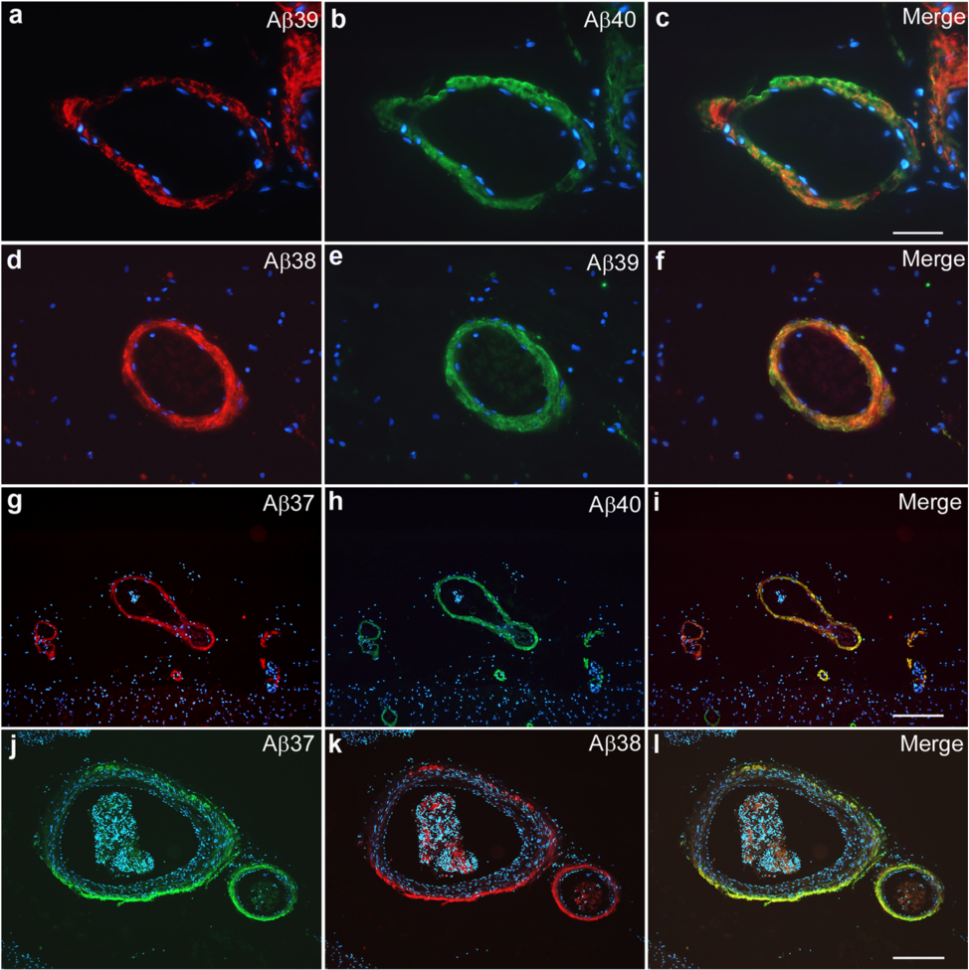
Vascular immunoreactivity against C-terminal truncated Aβ peptides in SAD. While Aβ38 showed an overlapping staining profile with Aβ37 (**j-l**) and Aβ39 (**d**-**f**), both peptides showed only a partial co-localization with Aβ40 (**a**-**c**, **g**-**i**). Scale bar: **a**-**c**, **g**-**l**: 200 μm; **d**-**f**: 50 μm

Aβ37 and Aβ39 immunoreactivity was detected in the majority of sporadic AD brains investigated (8 of 13 each) (Table 1; Fig. 1). Like Aβ38 and Aβ40, both peptides were mainly present in the vasculature of those cases presenting with abundant CAA. In contrast, none of 8 NDC subjects presented Aβ37 and Aβ39 immunoreactivity in parenchyma or vessels. Unlike Aβ40, the shorter peptide species Aβ37, Aβ38 and Aβ39 were scarcely detected within amyloid plaques, although considerable plaque pathology was present in all sporadic AD, as well as some non-demented control cases, as shown by 4G8 immunohistochemistry (Table 1). In general, both meningeal and parenchymal vessels were stained. However, in some cases meningeal vessels showed a more prominent immunoreactivity compared to parenchymal vessels (Fig. 1). In order to evaluate whether Aβ37 and Aβ39 co-localized with Aβ40, the major Aβ peptide species found in vascular amyloid, double immunofluorescence analysis was carried out. While vascular deposition of Aβ37 was found to be co-localized with Aβ40 in most cases, Aβ39 was found to exhibit a distinct distribution which differed from the Aβ40 staining pattern within the majority of vessels. In addition, Aβ40 staining was observed in many vessels which were neither stained with antibodies against Aβ37 nor Aβ39, arguing against relevant cross-reactions of the antibodies used in the current studies (Fig. 1). In the DS case # 18 showing Aβ39 immunoreactivity, staining was mainly present in large meningeal vessels together with Aβ37, while both were absent from parenchymal vessels. In contrast, Aβ38 was found to be abundantly present in both meningeal and parenchymal vessels. In the cases with AD + CAA, Aβ37, Aβ38, Aβ39 and Aβ40 were abundant in parenchymal and meningeal vessels, while in case #15 also faint Aβ39 immunoreactivity was detected in extracellular deposits (Additional file 1: Figure S1).

**Table 1:** Clinical and pathological data of sporadic AD cases and non-demented controls. Aβ staining intensity: - no staining; (+) barely detectable staining, + weak staining, ++ moderate staining, +++ abundant staining; *NP* neuritic plaques

						4G8		Aβ37		Aβ38		Aβ39		Aβ40	
No.	Age	Sex	Braak	Diagnosis	ApoE	NP	CAA	NP	CAA	NP	CAA	NP	CAA	NP	CAA
#1	92	M	IV	AD	3/3	++	+	-	-	-	+	-	+	+	+
#2	92	F	IV	AD	3/3	+	++	-	-	-	+	-	-	+	+
#3	93	M	IV	AD	3/3	+	+++	-	+++	-	++	-	++	++	+++
#4	91	M	IV	AD	4/2	++	+++	-	+	-	+	-	-	++	+++
#5	84	F	IV	AD	4/3	++	+	-	+	-	++	-	++	+	++
#6	91	F	IV	AD	4/3	++	++	-	++	(+)	+	-	+	++	+++
#7	88	F	IV	AD	3/3	+	+	-	-	-	-	-	-	+	+
#8	92	F	IV	AD	4/2	++	++	-	+	(+)	+	-	+	+	++
#9	79	F	IV	AD	4/3	++	+++	(+)	++	(+)	+++	-	++	++	+++
#10	84	F	IV	AD	3/2	++	-	-	-	-	-	-	-	+	-
#11	91	F	IV	AD	4/3	++	+	-	(+)	-	-	-	(+)	(+)	+
#12	86	M	IV	AD	3/3	+	++	-	+++	-	++	-	+++	+	++
#13	88	F	IV	AD	3/3	++	+	-	-	-	-	-	-	-	+
#14	96	F	V	AD + CAA	4/3	++	+++	-	+	-	+++	-	++	++	+++
#15	82	F	V	AD + CAA	3/3	++	+++	-	++	-	+++	+	+++	+++	+++
#16	61	F	VI	DS	3/3	++	-	-	-	-	-	-	-	+	-
#17	58	M	VI	DS	4/3	++	++	(+)	+	+	+	-	-	++	++
#18	64	F	V	DS	3/3	++	++	-	++	-	++	-	++	++	++
#19	91	M	I	NDC	3/3	-	-	-	-	-	-	-	-	-	-
#20	78	F	I	NDC	3/3	(+)	-	-	-	-	-	-	-	-	+
#21	73	M	0	NDC	3/3	-	-	-	-	-	-	-	-	-	-
#22	84	M	I	NDC	3/3	-	-	-	-	-	-	-	-	-	-
#23	88	F	I	NDC	3/3	+	-	-	-	-	-	-	-	-	-
#24	78	M	I	NDC	4/3	++	+	-	-	-	+	-	-	+	++
#25	82	F	I	NDC	3/3	-	-	-	-	-	-	-	-	-	-
#26	70	M	0	NDC	3/2	-	-	-	-	-	-	-	-	-	-

### Familial AD

**Fig. 2:**
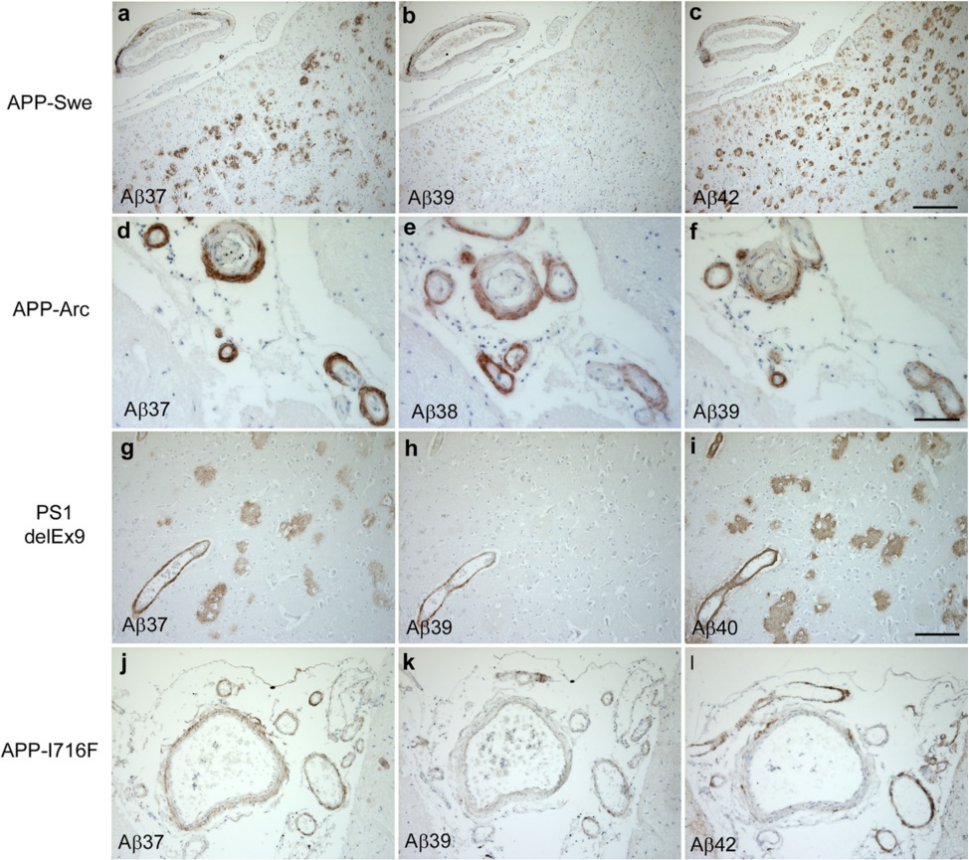
Vascular and parenchymal C-terminal truncated Aβ deposits were detected in FAD. Aβ37 and Aβ42 could be detected in parenchymal Aβ deposits, while Aβ39 showed only vascular immunoreactivity in an APP-Swe case (**a**-**c**). Abundant Aβ37-, Aβ38- and Aβ39-immunoreactivity could be demonstrated in meningeal vessels in an APP-Arc mutation carrier (**d**-**f**). A case with the PSEN ΔEx9 mutation showed robust Aβ37- and Aβ40-positive extracellular deposits, while Aβ39-immunoreactivity was restricted to vascular compartments (**g**-**i**). A case with the APP I716F mutation revealed abundant Aβ37 vascular staining, but only limited Aβ39- and Aβ42-immunoreactivity (**j**-**l**). Scale bar: **a**-**c**, **g**-**l**: 100 μm; **d**-**f**: 50 μm

The analysis of FAD cases included three cases with different APP mutations and a case with the PSEN1 mutation ΔExon9 (Table 2). In the latter, abundant parenchymal plaques were detected, that were stained by Aβ40 as well as by Aβ37 and Aβ38. In contrast, Aβ39 immunoreactivity was only present within the vasculature but not in extracellular deposits (Fig. 2g-i).

**Table 2:** Familial AD cases analyzed in the present study. Aβ staining intensity: - no staining; (+) barely detectable staining, + weak staining, ++ moderate staining, +++ abundant staining, *n.a* not analyzed, *NP* neuritic plaques

				Aβ37		Aβ38		Aβ39		Aβ40		Aβ42	
	Age	Sex	Mutation	NP	CAA	NP	CAA	NP	CAA	NP	CAA	NP	CAA
PS1-ΔEx9	61	m	ΔExon9	++	++	+	+	-	+	++	++	+++	(+)
APP-I716F	47	m	I716F	++	++	+	++	-	(+)	++	++	+++	+
APP-Arctic	64	m	E693Q	++	+++	+	++	+	++	+++	++	+++	(+)
APP-Swe	61	f	KM670/671NL	+	++	-	++	-	+	n.a.	n.a.	+++	(+)

In one case with the KM670/671NL APP (Swedish) mutation, a mutation located in the immediate vicinity of the β-secretase cleavage site and which has been described to increase total Aβ production [33], Aβ39 staining was found to be mainly limited to the vasculature, while Aβ37 could be detected within extracellular plaques as well (Fig. 2a, b).

The patient with the E693G APP (Arctic) mutation displayed severe amyloid plaque pathology, as previously described [23]. Of the four FAD mutation brains investigated, it was the only case to show Aβ39 immunoreactivity within extracellular amyloid deposits, presumably due to the overall enhancement of aggregation caused by this intra-Aβ-coding region mutation of APP [34] (Fig. 2).

The investigated FAD cases further included a case of the recently described APP mutation I716F [14, 46]. To the best of our knowledge, it was the first time that tis mutation was analyzed with respect to the brain deposition of Aβ species with varying C-termini. While Aβ42 was the predominant species within amyloid plaques, Aβ40 and the C-terminally truncated species Aβ37 and Aβ38 were mainly present within the vasculature, predominantly in larger meningeal vessels (Fig. 2). In the hippocampal region Aβ37, Aβ38 and Aβ40 contributed to plaque pathology as well, while Aβ39 did not appear within amyloid deposits and was only faintly detected in some of the vessels.

### Transgenic mouse models

**Fig. 3:**
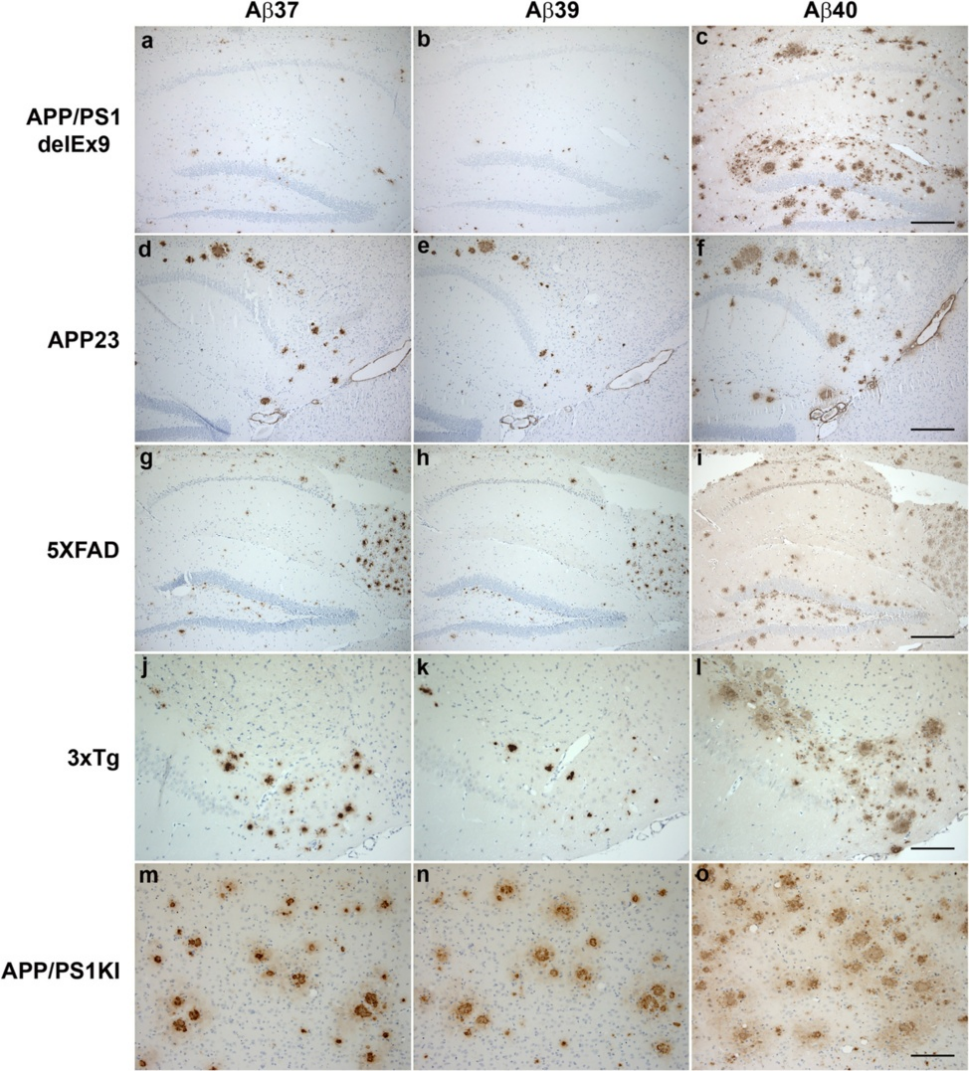
Immunohistochemical analysis of Aβ37, Aβ39 and Aβ40 in APP/PS1delEx9 (**a**-**c**), APP23 (**d**-**f**), 5XFAD (**g**-**i**), 3xTg (**j**-**l**) and APP/PS1KI mice (**m**-**o**). All peptides were detectable within extracellular deposits to a varying extent depending on the transgenic model. Scale bar: **a**-**i**: 200 μm; **j**-**o**: 100 μm

**Fig. 4:**
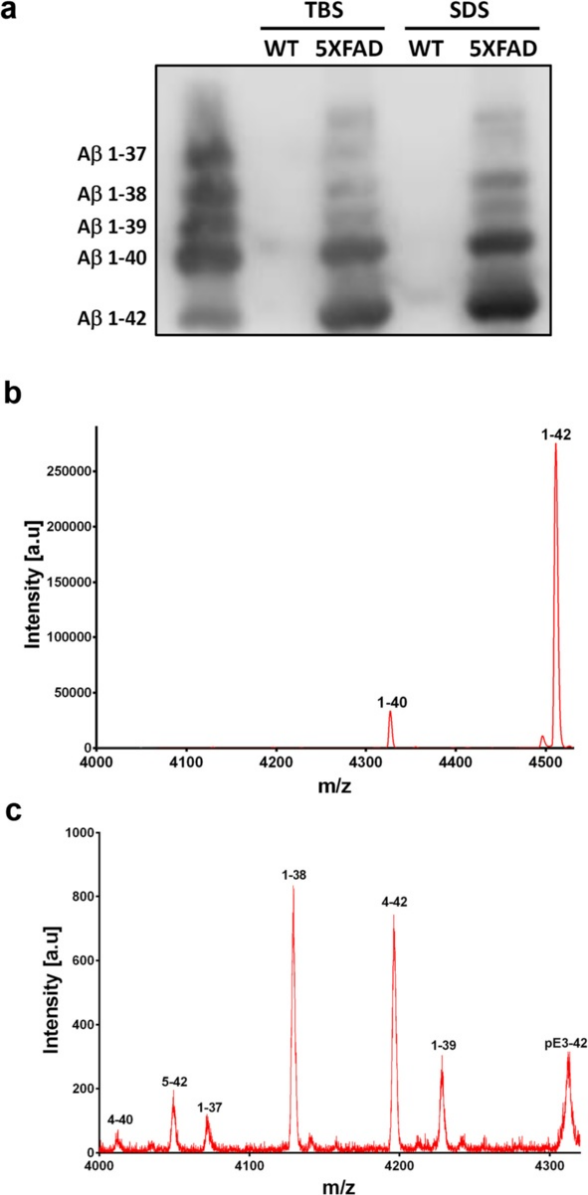
C-terminal heterogeneity of Aβ peptides in 5XFAD mice. Immunoprecipitation using 6E10 followed by detection with 1E8 demonstrates the presence of C-terminal truncated Aβ species in both TBS- and SDS-soluble fractions from aged 5XFAD brain lysates (**a**). Mass spectra of immunoprecipitated Aβ peptides using pan Aβ antibodies 6E10 and 4G8 (used as a mix). The dominant Aβ species in 5XFAD is Aβ1-42, followed by Aβ1-40 (**b**), while the C-terminal truncated species Aβ1-37, Aβ1-38 and Aβ139 could be detected in lower abundance (**c**)

We also investigated several established transgenic AD mouse models for the deposition of C-terminally truncated Aβ peptides by immunohistochemistry (Table 3). In contrast to human AD cases, all of the investigated transgenic AD mouse models exhibited Aβ37 and Aβ39 extracellular amyloid pathology to a varying degree, while vascular Aβ37 and Aβ39 immunoreactivity is almost absent. Our study suggests that plaques in AD mouse models contain a diverse spectrum of Aβ peptides of varying C-termini, including the C-terminally truncated Aβ species Aβ37, 38 and 39 (Fig. 3). 5XFAD and APP/PS1KI mice showed the most abundant overall extracellular amyloid plaque pathology and also the strongest staining for Aβ37 and Aβ39. Concordantly, C-terminal truncated Aβ species were detected in brain tissue lysates of 5XFAD mice using 1E8 antibody in a western blot performed following immunoprecipitation with 6E10 and Urea SDS-PAGE (Fig. 4a). Brain tissue from 7-month-old heterozygous 5XFAD mice was further analyzed by mass spectrometry. In addition to several N-terminal truncated Aβ species, Aβ1-37, Aβ1-38 and Aβ1-39 were identified, albeit less abundant than Aβ1-40 and Aβ1-42, which corroborates the immunohistochemical analyses (Fig. 4b, c). Double immunofluorescence of Aβ37 and Aβ39 with Aβ40 revealed major co-localization in extracellular plaques in 7-month-old 5XFAD and 10-month-old APP/PS1KI mice (Additional file 1: Figure S2).

**Table 3:** Extracellular Aβ-pathology in transgenic AD mouse models. Aβ staining intensity: + weak staining, ++ moderate staining, +++ abundant staining, *n.a* not analyzed

Transgenic model	Age	Aβ37	Aβ38	Aβ39	Aβ40
APP/PS1ΔEx9	9 m	+	++	+	+++
5XFAD	7 m	+++	+++	+++	+++
PDAPP	18 m	+	+	+	++
APP23	20 m	++	n.a.	++	+++
3xTg	18 m	++	++	++	+++
APP/PS1KI	10 m	+++	+++	+++	+++

## Discussion

Even though research has long been centered on Aβ peptides and reports on the accumulation of Aβ40 and Aβ42 are numerous, the deposition of the C-terminally truncated Aβ peptides shorter than 40 amino acids in sporadic and familial AD patients has not been thoroughly investigated in post-mortem tissue. Recent studies by Moro and colleagues [32], as well as by our group [44], reported Aβ38 to be abundantly deposited within the vasculature of SAD cases presenting severe CAA, as well as in various FAD cases with underlying APP and PSEN1 mutations. To the best of the authors’ knowledge, there are currently no studies available describing the immunohistochemical analysis of the C-terminally truncated species Aβ37 or Aβ39 in human AD cases or transgenic mouse models. In previous biochemical studies, both peptides have been shown to be detectable within human CSF and blood plasma samples [27, 55] or neuroblastoma cells [1]. Mass spectrometry characterization of human brain samples failed to detect Aβ1-39 in SAD and only faintly detected Aβ1-37 in one out of five cases investigated. In contrast, both subtypes were present in one out of three investigated cases with KM670/671NL (Swedish) mutation [41]. In another recent study, Aβ1-37 and Aβ1-39 were identified in a small fraction of AD and cases of pathological aging but not in non-demented control samples [31]. Prelli and colleagues reported already in 1988 that cerebrovascular Aβ, although homologous to plaque core amyloid, consists of only 39 instead of 42 amino acid residues [42]. This finding has been partially confirmed by Miller et al., who detected mainly Aβ1-40 with a minor proportion of Aβ1-39 and Aβ2-39 in an analysis of cerebrovascular amyloid [30].

In the present study, we found the two C-terminally truncated Aβ species Aβ37 and Aβ39 to be present in vascular compartments in the majority of analyzed SAD cases and in one NDC case. The amount of deposits corresponded to the variable degree of CAA in the analyzed cases, as revealed by 4G8 staining, and was generally correlated with the deposition of Aβ38.

The observed pattern of deposition confirms in vitro analyses of Aβ peptides with varying C-termini that rendered the C-terminally truncated species to be rather soluble and less prone to aggregation when compared to Aβ42 [22, 52]. Although the C-terminally truncated Aβ species seem to have similar aggregation propensities as Aβ40, they are considerably less abundant within parenchymal Aβ deposits of SAD cases. This is most likely caused by a lower production rate of the C-terminally truncated peptides compared to Aβ40 production. While Aβ40 typically accounts for over 50 % of Aβ production, C-terminally truncated variants of Aβ (Aβ1-37, Aβ 1-38 and Aβ 1-39), as well as the more toxic Aβ42 species, have been found to account for only minor fractions of the total Aβ production in most cells [3, 12, 38]. It has been shown that even subtle changes in the proportion between different Aβ species can facilitate disease progression. In this respect, the Aβ42/40 ratio may be of particular significance as Aβ40 has been found to hinder the aggregation of Aβ42 [24, 25]. This is also underscored by a recent report showing that Aβ1-40 plays a different role in tau pathogenesis compared to Aβ1-42. It has been speculated that Aβ1-40 may have a protective role in tau pathogenesis by reducing phosphorylation at Ser262, an epitope that renders tau to be more neurotoxic [20].

Recent findings of a vast decrease of Aβ1-37, Aβ1-38 and Aβ1-39 in the CSF of FAD cases have prompted speculation upon a similar protective function of the C-terminally truncated Aβ species, that was absent in FAD due to reduced production rates [40]. On the other hand, using double-immunofluorescence staining of SAD cases, we found Aβ39 to exhibit a virtually opposite deposition pattern as Aβ40, while Aβ37 was found to be mostly co-localized with Aβ40.

In different cases of FAD we found Aβ37 and Aβ39, which had been limited to the vasculature in SAD, to contribute to NPs as well. We chose different APP mutations, located at the N-terminal (APP_Swe_), the mid-portion (APP_Arc_) and the C-terminal part of Aβ (APP_I716F_), in order to allow conclusions about the mechanisms involved that lead to deposition of the analyzed peptides. Interestingly Aβ39 was only detected within NPs in a case carrying the intra-Aβ E693G mutation, a mutation that has been described to strongly enhance aggregation propensities and hinder proteolytic degradation of Aβ [34, 51]. Conversely Aβ37 was detected to a varying degree in NPs of all analyzed FAD cases. This might be due to an increase in Aβ42 levels in APP mutation carriers, as multiple pathways for stepwise successive γ-secretase cleavages, including the release of the GVVIA pentapeptide from Aβ42, have been proposed [29]. Presenilin mutations have been demonstrated to change the spectrum of Aβ species produced by γ-secretase and to increase the Aβ42/Aβ40 ratio, mainly by lowering Aβ40 production [7].

The I716F APP mutation represents a mutation in close proximity to the ε-cleavage site of γ-secretase [14, 46]. The recent discovery of I716F verified earlier in vitro experiments predicting that the mutation would cause extraordinarily severe Aβ accumulation [26]. There is ample evidence showing that the I716F APP mutation causes drastic elevations of Aβ42 production in vitro, with a dramatic increase in the Aβ42/Aβ40 ratio [19, 26]. This was interpreted as a shift in Aβ product lines due to impaired ε-cleavage of γ-secretase. Thereby the mutation would immensely enhance the product line starting with Aβ48 (Aβ48, Aβ45, Aβ42, Aβ38), while suppressing the product line starting with Aβ49 (Aβ49, Aβ46, Aβ43, Aβ40) [49]. In good agreement with these findings, we found Aβ42 to be the predominant species deposited in the brain of a case carrying this mutation. On the other hand, significant amounts of Aβ40 and Aβ37 were detected as well, underscoring their dependence in terms of production, while the relative abundance of Aβ37 may also underline the significance of recently described deviations from the major product lines of γ-secretase [29].

The analysis of a group of widely used transgenic AD mouse models revealed Aβ37 and Aβ39 to be widely present to a varying extent within NPs to a degree depending on the overall severity of plaque pathology in each model. Vascular Aβ37 and Aβ39 immunoreactivity is almost absent, which might be due to the fact that most models in general harbor only minor Aβ vessel pathology. Mass spectrometry analysis in 5XFAD mice revealed that C-terminal truncated Aβ variants only represent a minor proportion. However their presence might represent a potential read-out to measure the effect of different γ-secretase modulators in vivo.

## Conclusion

Taken together, our study points out that a broad range of C-terminally modified Aβ peptides are present within the vasculature of SAD and FAD patients. Some of these peptide variants were also detected within the extracellular Aβ deposits in FAD patients, while all analyzed transgenic mouse models based on FAD causing mutations harbor Aβ37-, Aβ38- and Aβ39-positive plaques to a varying degree. The exact role of these C-terminally truncated peptides within the vasculature and the underlying mechanisms responsible for their heterogeneity have to be elucidated in future studies.

## Additional file

Additional file 1: Deposition of C-terminally truncated Aβ species Aβ37 and Aβ39 in Alzheimer’s disease and transgenic mouse models. **Figure S1.** Immunohistochemical detection of Aβ37, Aβ38, Aβ39 and Aβ40 in a case with AD and CAA. While Aβ37 (a) and Aβ38 (b) immunoreactivity is restricted to vascular compartments, Aβ39 (c) and Aβ40 (d) are detectable within vessels, as well as parenchymal extracellular amyloid deposits. Scale bar: a-d: 100 μm. **Figure S2.** Aβ37 and Aβ39 peptide species are co-localized with Aβ40 in extracellular amyloid deposits in the cortex of 7-month-old 5XFAD mice (a), as well as 10-month-old APP/PS1KI mice (b). Scale bar: a: 50 μm; b: 100 μm. (PDF 700 kb)
