# Supplementary material for: Deposition of C-terminally truncated Aβ species Aβ37 and Aβ39 in Alzheimer’s disease and transgenic mouse models
Source: Acta Neuropathol Commun. 2016 Mar 8;4:24. doi: 10.1186/s40478-016-0294-7 (PMC4784385; doi:10.1186/s40478-016-0294-7)
Supplement: Additional file 1: — Deposition of C-terminally truncated Aβ species Aβ37 and Aβ39 in Alzheimer’s disease and transgenic mouse models. Figure S1. Immunohistochemical detection of Aβ37, Aβ38, Aβ39 and Aβ40 in a case with AD and CAA. While Aβ37 (a) and Aβ38 (b) immunoreactivity is restricted to vascular compartments, Aβ39 (c) and Aβ40 (d) are detectable within vessels, as well as parenchymal extracellular amyloid deposits. Scale bar: a-d: 100 μm. Figure S2. Aβ37 and Aβ39 peptide species are co-localized with Aβ40 in extracellular amyloid deposits in the cortex of 7-month-old 5XFAD mice (a), as well as 10-month-old APP/PS1KI mice (b). Scale bar: a: 50 μm; b: 100 μm. (PDF 700 kb) [file 40478_2016_294_MOESM1_ESM.pdf]

Supplemental Information: Reinert et al.: „Deposition of C-terminally truncated Aβ species Aβ37 and Aβ39 in Alzheimer's disease and transgenic mouse models"

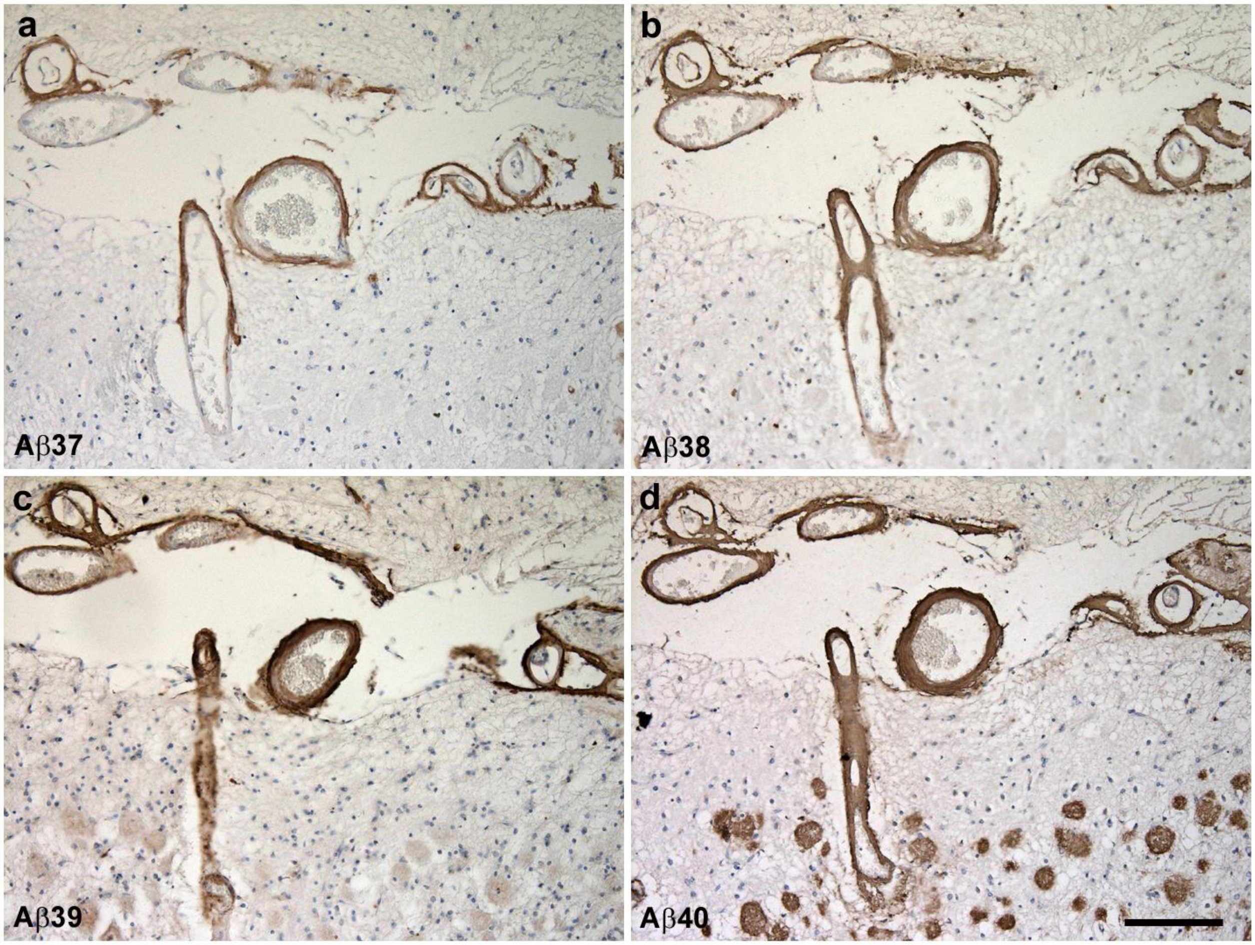

Supplemental Fig. 1:

Immunohistochemical detection of Aβ37, Aβ38, Aβ39 and Aβ40 in a case with AD and CAA. While Aβ37 (**a**) and Aβ38 (**b**) immunoreactivity is restricted to vascular compartments, Aβ39 (**c**) and Aβ40 (**d**) are detectable within vessels, as well as parenchymal extracellular amyloid deposits. Scale bar: a-d: 100 µm.

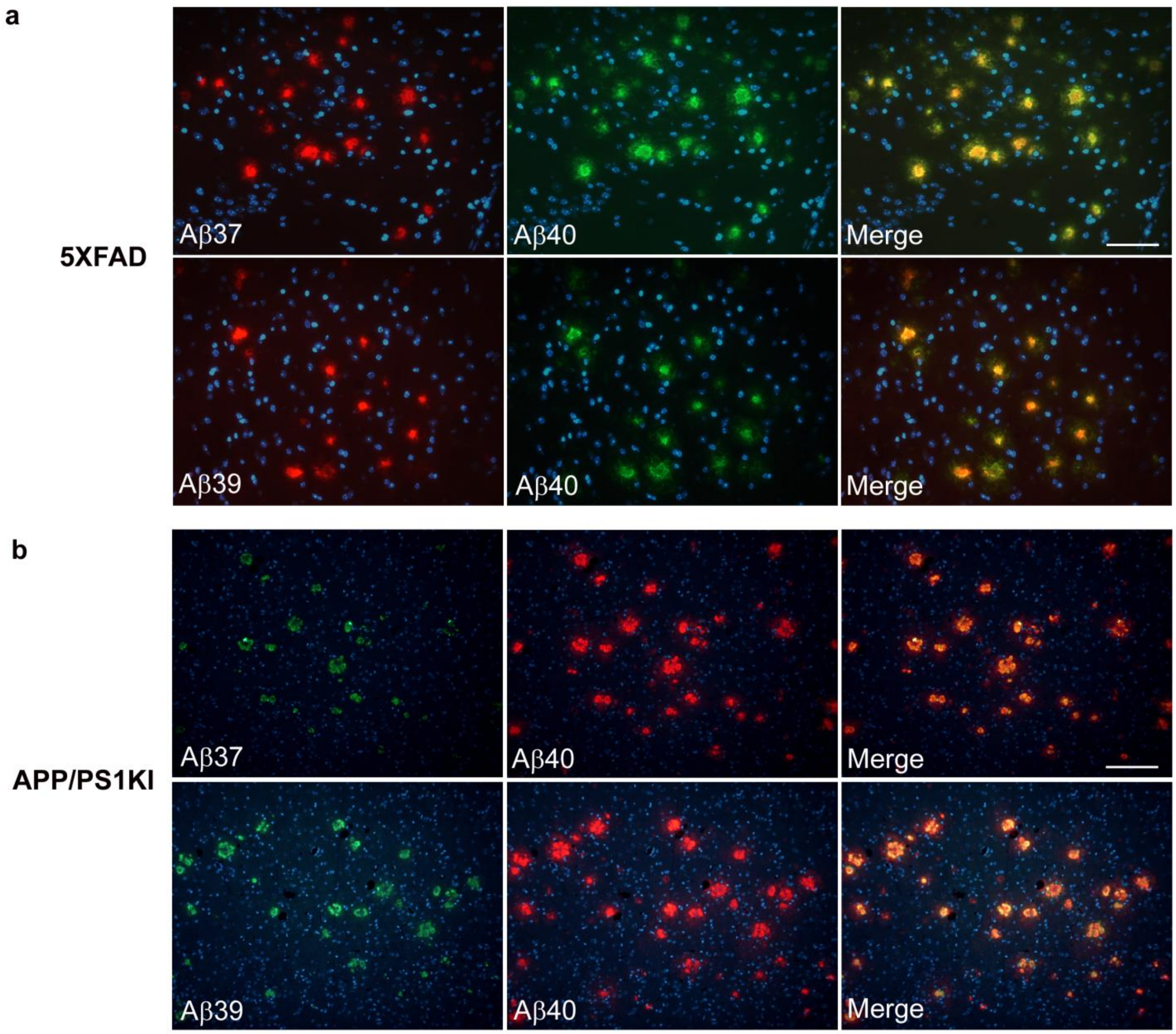

Supplemental Fig. 2:

Aβ37 and Aβ39 peptide species are co-localized with Aβ40 in extracellular amyloid deposits in the cortex of 7-month-old 5XFAD mice (**a**), as well as 10-month-old APP/PS1KI mice (**b**). Scale bar: a: 50 µm; b: 100 µm.
